# Clinical Characteristics and Predictors of Hypokalemia in Patients Receiving Piperacillin/Tazobactam: A Retrospective Observational Audit

**DOI:** 10.7759/cureus.103408

**Published:** 2026-02-11

**Authors:** Taonga Gogo-Peters, Musa Usman Nahuche, Somtochukwu Okafor

**Affiliations:** 1 Medicine, Mater Dei Hospital, Msida, MLT; 2 Geriatrics, Mater Dei Hospital, Msida, MLT; 3 Emergency Medicine, Mater Dei Hospital, Msida, MLT

**Keywords:** clinical pharmacology, drug-induced hypokalemia, drug-related side effects and adverse reactions, electrolyte disturbance, inpatient unit, piperacillin/tazobactam, retrospective observational study

## Abstract

Hypokalemia is a clinically relevant electrolyte disturbance that may develop during treatment with piperacillin/tazobactam. Although often described as uncommon, emerging clinical experience suggests that the true incidence may be higher, particularly among hospitalized patients receiving prolonged therapy. This retrospective observational audit evaluated the incidence, severity, and risk factors associated with hypokalemia in adult inpatients treated with piperacillin/tazobactam at Mater Dei Hospital. Medical records of 62 adult patients who received piperacillin/tazobactam for at least three consecutive days between May 1, 2024, and August 31, 2024, were reviewed. Serum potassium values, demographic data, duration of therapy, comorbidities, and potassium supplementation patterns were examined. Hypokalemia developed progressively with treatment duration, with the highest incidence observed around Day 5 of therapy. Female patients and those with baseline potassium levels below 4.0 mmol/L demonstrated a greater predisposition to developing hypokalemia. Although hypokalemia was observed during therapy, potassium supplementation generally led to improvement in serum potassium levels, indicating that supplementation was effective in supporting biochemical recovery. Higher initial potassium levels appeared to be protective, highlighting the importance of early risk assessment and optimization of baseline potassium. This analysis emphasizes that hypokalemia is a relatively common occurrence in patients treated with piperacillin/tazobactam in routine clinical practice. The findings support the need for proactive monitoring, identification of high-risk groups, and preventive strategies, such as optimizing baseline potassium and closer surveillance during the first week of therapy, to reduce the likelihood of clinically significant electrolyte disturbances.

## Introduction

Hypokalemia is a common and clinically significant electrolyte abnormality that can result in muscle weakness, gastrointestinal dysfunction, and potentially life-threatening cardiac arrhythmias if not promptly recognized and managed [1]. Medication-induced disturbances in potassium homeostasis are well documented, and broad-spectrum antibiotics are an important, though often underrecognized, contributor to this condition [2].

Piperacillin/tazobactam is widely prescribed for moderate to severe infections due to its broad antimicrobial coverage and favorable clinical profile. Although generally considered safe, emerging evidence suggests that it may be associated with a higher-than-expected incidence of hypokalemia. Previous studies have reported incidence rates ranging from 12.6% to 24.8%, indicating that this risk may be more clinically relevant than traditionally assumed [3-5]. Proposed mechanisms include renal potassium wasting due to the drug’s non-resorbable anion effect within the distal nephron, which promotes increased urinary potassium excretion [6].

Prescribing guidance, including the summary of product characteristics, underscores the importance of recognizing potential electrolyte disturbances associated with piperacillin/tazobactam therapy [7]. Certain patient groups may be more vulnerable, such as older adults, females, and individuals with low baseline potassium levels. Early identification of these risk factors and appropriate monitoring may help reduce treatment interruptions and prevent complications [4,5,8].

At Mater Dei Hospital, piperacillin/tazobactam is used extensively across multiple departments, yet its impact on potassium homeostasis has not been previously characterized in the local population. Understanding the incidence, timing, and severity of hypokalemia associated with its use may support safer prescribing practices and guide the implementation of proactive monitoring strategies, in line with international pharmacovigilance principles [9].

This retrospective observational audit was therefore undertaken to determine the incidence and severity of hypokalemia in patients treated with piperacillin/tazobactam at our institution and to identify potential risk factors contributing to its development.

## Materials and methods

This study was designed as a retrospective observational audit conducted at Mater Dei Hospital, Malta. The objective of the audit was to evaluate the incidence, severity, onset pattern, and potential risk factors associated with hypokalemia in adult inpatients treated with piperacillin/tazobactam.

Medical records of adult patients aged 18 years and older who received piperacillin/tazobactam for a minimum of three consecutive days were reviewed. Data were collected retrospectively from May 1, 2024, to August 31, 2024, a period during which piperacillin/tazobactam was frequently prescribed across multiple medical wards.

Patients were eligible for inclusion if they had received piperacillin/tazobactam for at least three days and had documented serum potassium measurements prior to initiation of therapy, during treatment, and at the time of antibiotic discontinuation. Patients were excluded if baseline serum potassium measurements were unavailable; if baseline hypokalemia or hyperkalemia was present due to unrelated clinical conditions; if they were receiving concurrent medications known to significantly reduce serum potassium levels, such as potassium-wasting loop diuretics; if they had active clinical conditions that independently affected potassium homeostasis; or if incomplete laboratory data prevented adequate analysis of potassium trends. These exclusion criteria were applied to ensure that observed changes in potassium levels could be reasonably attributed to piperacillin/tazobactam therapy rather than confounding factors [7,8].

Data were extracted from both electronic and paper-based medical records. Collected variables included patient age and sex, relevant comorbidities, baseline serum potassium levels, serial serum potassium measurements during antibiotic therapy, the duration and dosing regimen of piperacillin/tazobactam, and the use of potassium supplementation. Serum potassium levels were recorded on treatment Days 1, 3, 5, 7, and 10 when available. Hypokalemia was classified according to severity: mild hypokalemia was defined as serum potassium levels between 3.0 and 3.5 mmol/L, moderate hypokalemia as levels between 2.5 and 3.0 mmol/L, and severe hypokalemia as levels below 2.5 mmol/L. These thresholds are consistent with established clinical criteria for potassium disorders [5,6].

The primary outcomes of interest were the incidence of hypokalemia on each treatment day and the distribution of hypokalemia severity among affected patients. Secondary outcomes included the relationship between baseline potassium levels and the subsequent development of hypokalemia, differences in susceptibility based on age and sex, and the progression of potassium decline over the duration of antibiotic therapy.

Potassium supplementation was administered at the discretion of the treating clinical teams following routine blood investigations that identified hypokalemia. Supplementation practices were not standardized, and the timing, dose, route, and biochemical response to supplementation were not systematically analyzed.

The audit was primarily descriptive in nature. Data analysis was performed using descriptive statistical methods, including calculation of frequencies, percentages, means, and ranges where appropriate. No inferential statistical tests were conducted, as the audit was designed as a quality improvement study.

## Results

A total of 62 adult patients treated with piperacillin/tazobactam met the inclusion criteria and were included in the analysis. The study population comprised 25 female patients (40.3%) and 37 male patients (59.7%). Twelve patients (19.4%) were aged 65 years or younger, while 50 patients (80.6%) were older than 65 years. Baseline demographic characteristics of the study population are summarized in Table 1.

**Table 1:** Incidence and severity of hypokalemia during piperacillin/tazobactam therapy This table summarizes the occurrence and severity of hypokalemia among adult inpatients treated with piperacillin/tazobactam, both overall and stratified by age, sex, and baseline serum potassium level. For each treatment day, the denominator (N) represents the number of patients actively receiving piperacillin/tazobactam with available serum potassium measurements on that day. The total number of patients decreases across time points due to antibiotic discontinuation, hospital discharge, death, or missing laboratory data. Total hypokalemia events represent the number of patients with serum potassium <3.5 mmol/L at each time point. Hypokalemia severity was classified as mild (3.0-3.5 mmol/L), moderate (2.5-3.0 mmol/L), or severe (<2.5 mmol/L). Percentages were calculated using the number of patients at risk on the corresponding day as the denominator. Subgroup analyses (age ≤65 years vs. >65 years, female vs. male, baseline potassium <4.0 mmol/L vs. ≥4.0 mmol/L) followed the same approach, with denominators varying by subgroup and time point.

Patient group (N)	Total hypokalemia events, N (%)	Mild hypokalemia (3.0-3.5 mmol/L), N (%)	Moderate hypokalemia (2.5-3.0 mmol/L), N (%)	Severe hypokalemia (<2.5 mmol/L), N (%)
Overall cohort				
Day 1 Tazocin (N = 62)	6 (9.7%)	6 (9.7%)	0 (0%)	0 (0%)
Day 3 Tazocin (N = 53)	12 (22.6%)	10 (18.9%)	1 (1.9%)	1 (1.9%)
Day 5 Tazocin (N = 47)	14 (29.8%)	10 (21.3%)	4 (8.5%)	0 (0%)
Day 7 Tazocin (N = 41)	10 (24.4%)	5 (12.2%)	4 (9.8%)	1 (2.4%)
Day 10 Tazocin (N = 38)	5 (13.2%)	2 (5.3%)	2 (5.3%)	1 (2.6%)
Patients ≤65 years (N = 12)				
Day 1 (N = 12)	0 (0%)	0 (0%)	0 (0%)	0 (0%)
Day 3 (N = 9)	1 (11.1%)	1 (11.1%)	0 (0%)	0 (0%)
Day 5 (N = 7)	2 (28.6%)	0 (0%)	2 (28.6%)	0 (0%)
Day 7 (N = 6)	1 (16.7%)	1 (16.7%)	0 (0%)	0 (0%)
Day 10 (N = 5)	1 (20.0%)	0 (0%)	1 (20.0%)	0 (0%)
Patients >65 years (N = 50)				
Day 1 (N = 50)	8 (16.0%)	6 (12.0%)	1 (2.0%)	1 (2.0%)
Day 3 (N = 43)	11 (25.6%)	9 (20.9%)	1 (2.3%)	1 (2.3%)
Day 5 (N = 38)	12 (31.6%)	10 (26.3%)	2 (5.3%)	0 (0%)
Day 7 (N = 33)	9 (27.3%)	4 (12.1%)	4 (12.1%)	1 (3.0%)
Day 10 (N = 30)	4 (13.3%)	2 (6.7%)	1 (3.3%)	1 (3.3%)
Female patients (N = 25)				
Day 1 (N = 25)	5 (20.0%)	4 (16.0%)	1 (4.0%)	0 (0%)
Day 3 (N = 23)	6 (26.1%)	5 (21.7%)	1 (4.3%)	0 (0%)
Day 5 (N = 18)	7 (38.9%)	5 (27.8%)	2 (11.1%)	0 (0%)
Day 7 (N = 16)	7 (43.8%)	5 (31.3%)	1 (6.3%)	1 (6.3%)
Day 10 (N = 13)	5 (38.5%)	2 (15.4%)	2 (15.4%)	1 (7.7%)
Male patients (N = 37)				
Day 1 (N = 35)	2 (5.7%)	2 (5.7%)	0 (0%)	0 (0%)
Day 3 (N = 28)	6 (21.4%)	5 (17.9%)	0 (0%)	1 (3.6%)
Day 5 (N = 27)	7 (25.9%)	5 (18.5%)	2 (7.4%)	0 (0%)
Day 7 (N = 24)	3 (12.5%)	0 (0%)	3 (12.5%)	0 (0%)
Day 10 (N = 23)	0 (0%)	0 (0%)	0 (0%)	0 (0%)
Baseline potassium <4.0 mmol/L (N = 16)				
Day 1 (N = 16)	7 (43.8%)	6 (37.5%)	1 (6.3%)	0 (0%)
Day 3 (N = 15)	8 (53.3%)	8 (53.3%)	0 (0%)	0 (0%)
Day 5 (N = 12)	6 (50.0%)	4 (33.3%)	2 (16.7%)	0 (0%)
Day 7 (N = 12)	3 (25.0%)	2 (16.7%)	1 (8.3%)	0 (0%)
Day 10 (N = 10)	1 (10.0%)	0 (0%)	1 (10.0%)	0 (0%)
Baseline potassium ≥4.0 mmol/L (N = 46)				
Day 1 (N = 46)	0 (0%)	0 (0%)	0 (0%)	0 (0%)
Day 3 (N = 37)	4 (10.8%)	2 (5.4%)	1 (2.7%)	1 (2.7%)
Day 5 (N = 33)	8 (24.2%)	6 (18.2%)	2 (6.0%)	0 (0%)
Day 7 (N = 28)	6 (21.4%)	3 (10.7%)	2 (7.1%)	1 (3.6%)
Day 10 (N = 26)	4 (15.4%)	2 (7.7%)	1 (3.8%)	0 (0%)

Overall incidence and timing of hypokalemia

The incidence of hypokalemia increased with the duration of piperacillin/tazobactam therapy. On Day 1 of treatment, hypokalemia was observed in six of 62 patients (9.7%). By Day 3, 12 of 53 patients (22.6%) were hypokalemic. The highest incidence occurred on Day 5, when 14 of 47 patients (29.8%) developed hypokalemia. On Day 7, hypokalemia persisted in 10 of 41 patients (24%), and by Day 10, five of 38 patients (13.2%) remained hypokalemic. These temporal trends are detailed in Table 1, demonstrating a progressive rise in hypokalemia incidence up to Day 5, followed by a decline thereafter.

Despite routine potassium supplementation, hypokalemia continued to occur throughout the treatment period, indicating an ongoing potassium-lowering effect associated with piperacillin/tazobactam therapy.

Severity of hypokalemia

Across all observed time points, mild hypokalemia was the most frequently observed severity category. On Day 5, mild hypokalemia was present in 10 of 47 patients (21.3%), while moderate hypokalemia was observed in four of 47 patients (8.5%). Severe hypokalemia was uncommon overall but was documented on Day 7 in one of 41 patients (2.5%) and on Day 10 in one of 38 patients (2.6%), despite potassium supplementation. The distribution of hypokalemia severity across treatment days is presented in Table 1.

Age-based differences

Age-stratified analysis revealed differences in early hypokalemia risk. Among patients aged 65 years or younger, no cases of hypokalemia were observed on Day 1 (zero of 12 patients, 0%). In contrast, among patients older than 65 years, hypokalemia occurred on Day 1 in six of 50 patients (12%). By Day 5, hypokalemia was observed in two of seven younger patients (28.6%) and in 10 of 38 older patients (26.3%). From Day 3 onward, both age groups demonstrated similar trends in potassium decline. These age-related patterns are summarized in Table 1. Although moderate and severe hypokalemia occurred slightly more frequently in older patients, the overall progression patterns between the two age groups were broadly comparable.

Sex-based differences

Female patients demonstrated consistently higher susceptibility to hypokalemia throughout the treatment period. On Day 5, hypokalemia was observed in seven of 18 female patients (38.8%), compared with seven of 27 male patients (25.9%). By Day 7, hypokalemia persisted in seven of 16 female patients (43.8%), whereas no male patients were hypokalemic at that time point (zero of 24 patients, 0%). On Day 10, hypokalemia remained present in five of 13 female patients (38.5%), while no cases were observed among male patients (zero of 23 patients, 0%). These sex-based differences are detailed in Table 1.

Effect of baseline serum potassium

Baseline serum potassium level appeared to be associated with the subsequent development of hypokalemia. Among patients with baseline potassium levels below 4.0 mmol/L, hypokalemia occurred on Day 3 in eight of 15 patients (53.3%) and on Day 5 in six of 12 patients (50%). In contrast, among patients with baseline potassium levels of 4.0 mmol/L or higher, hypokalemia occurred on Day 5 in six of 33 patients (18.2%) and did not exceed 25% at any time point. These findings are presented in Table 1.

Comorbidities

**Figure 1:**
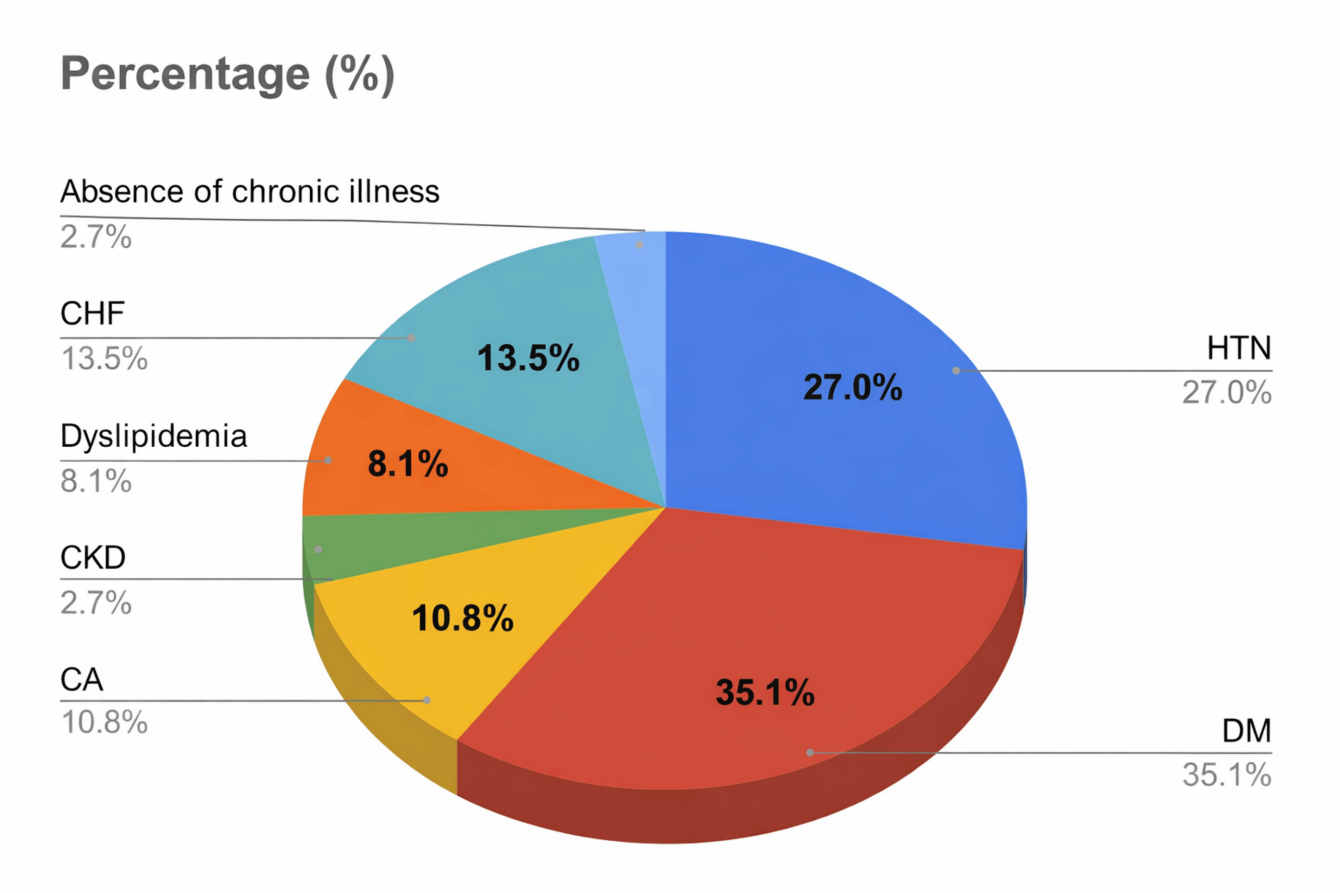
Distribution of comorbidities among patients who developed hypokalemia This figure illustrates the distribution of comorbid conditions among patients who developed hypokalemia at any point during piperacillin/tazobactam treatment. Comorbidity data correspond to the cohort of patients who experienced hypokalemia during the treatment course, with classification based on the presence of documented chronic medical conditions at the time hypokalemia was identified (peak hypokalemia incidence). Percentages are calculated using the total number of patients who developed hypokalemia (N = 37) as the denominator. Comorbidities included DM (13 patients, 35.1%), HTN (10 patients, 27.0%), CHF (five patients, 13.5%), CA (four patients, 10.8%), dyslipidemia (three patients, 8.1%), CKD (one patient, 2.7%), and absence of chronic illness (one patient, 2.7%). CA, cancer; CHF, congestive heart failure; CKD, chronic kidney disease; DM, diabetes mellitus; HTN, hypertension

The distribution of comorbidities among patients who developed hypokalemia during piperacillin/tazobactam therapy is shown in Figure 1. The comorbidity profile among affected patients closely reflected that of the overall study population, with no single condition demonstrating a disproportionate association with hypokalemia. This suggests that potassium decline during therapy was not driven by a specific comorbid profile.

## Discussion

This retrospective observational audit found that hypokalemia was a relatively common electrolyte abnormality among inpatients receiving piperacillin/tazobactam, with incidence increasing during the first week of therapy and peaking around Day 5. Similar temporal patterns have been reported in previous observational studies evaluating electrolyte disturbances associated with this antibiotic [3-5].

Hypokalemia appeared more frequently among female patients and those with lower baseline serum potassium levels. These findings represent descriptive observations within the study cohort and may help identify patient groups who warrant closer monitoring during therapy.

Age-related differences were observed primarily during the early treatment days, with hypokalemia occurring more frequently among older patients on Day 1. Beyond this early period, broadly similar patterns were observed across age groups. Given the descriptive design, no conclusions can be drawn regarding the relative contribution of age, comorbidity burden, or illness severity to these patterns.

The distribution of comorbidities among patients who developed hypokalemia was broadly similar to that of the overall study population. In the absence of inferential statistical analysis or a comparator group, these findings are presented descriptively and do not allow conclusions regarding the independent contribution of comorbidities to hypokalemia development.

Limitations

Several limitations should be acknowledged. As a retrospective observational audit, the analysis relied on the accuracy and completeness of existing medical records. Variability in the timing of serum potassium measurements and missing laboratory data may have influenced the observed incidence.

Hypokalemia was assessed through daily cross-sectional observations, and individual patients were not followed longitudinally. As a result, new-onset hypokalemia could not be distinguished from persistent hypokalemia at later treatment days.

The study was conducted at a single center with a relatively small sample size, which may limit generalizability. In addition, the absence of inferential statistical analysis and a comparator group precludes assessment of causality or independent predictors. Potential confounders, such as illness severity, renal function changes, intravenous fluid composition, and nutritional status, were not independently controlled for.

## Conclusions

Hypokalemia was a frequent and clinically relevant electrolyte disturbance among inpatients treated with piperacillin/tazobactam. It appeared more common among female patients and those with lower baseline serum potassium levels. The incidence increased during the first week of therapy, peaking around Day 5. These findings underscore the importance of baseline potassium assessment and close electrolyte monitoring during piperacillin/tazobactam treatment, particularly during the early phase of therapy.
